# Atypical Presentation of Rheumatoid Arthritis With Erythema Nodosum Mimicking Cellulitis: A Case Report and Literature Review of Erythema Nodosum

**DOI:** 10.1002/ccr3.70566

**Published:** 2025-06-02

**Authors:** Laxman Wagle, Parmartha Basnyat, Anuj Timshina, Rashmita Regmi

**Affiliations:** ^1^ Department of Internal Medicine Ascension Saint Agnes Hospital Baltimore Maryland USA; ^2^ Department of Internal Medicine Patan Academy of Health Science Kathmandu Nepal; ^3^ Department of Nursing Karnali Academy of Health Science Jumla Nepal

**Keywords:** anti‐cyclic citrullinated peptide antibody, erythema nodosum, Miescher's granuloma, rheumatoid arthritis, steroid therapy

## Abstract

Erythema nodosum (EN) can be an atypical manifestation of rheumatoid arthritis (RA), as seen in this case of a 35‐year‐old woman initially misdiagnosed with cellulitis. Despite negative cultures and antibiotic treatment, further evaluation revealed elevated rheumatoid factor and anti‐CCP antibodies, confirming RA. Steroids led to symptom resolution.

## 1 Introduction

Rheumatoid arthritis (RA) is a chronic inflammatory systemic disease, including systemic lupus erythematosus, that can involve multiple organ systems and present with diverse clinical features [1]. As per the Global Burden of Diseases, Injuries, and Risk Factors 2017 study, RA's age‐standardized point prevalence (number of people per 100,000 people) was 246.6 (95% UI 222.4–270.8) [2]. Similarly, it is also one of the major contributors to global disability [3]. Rheumatoid arthritis develops due to genetic predispositions, environmental triggers, and immunological factors, which lead to synovial immunological response, inflammation, and joint damage [4]. Typically, RA manifests with involvement of multiple joints but can also have atypical or extra‐articular involvement, including skin (rheumatoid nodules), heart, lungs (interstitial lung disease), cardiac (pericardial effusion), anemia, Felty syndrome, amyloidosis, carpal tunnel syndrome, scleritis, and many others.

These extra‐articular manifestations are seen in 17.8%–40.9% of the patients with RA [5]. It can have various cutaneous lesions ranging from rheumatoid nodules to vasculitis and dermatitis [6]. Cutaneous lesions are common but mostly present with symmetric arthritis [7]. Erythema nodosum (EN) is a septal panniculitis that results from a reactive process to various etiologies, such as inflammation, infections, neoplasms, and medications. Very few cases of EN have been reported in patients with RA. Streptococcal infection, primary tuberculosis, sarcoidosis, Behçet's disease, medication, inflammatory bowel disease, non‐Hodgkin lymphoma, and pregnancy were reported as common causes of EN. Still, no etiology was identified in several cases [8, 9, 10, 11, 12, 13, 14]. EN presents as painful, erythematous nodules in the subcutaneous tissue, usually seen on the shins. Histologically, it reveals septal panniculitis without vasculitis with early lesions showing lymphohistiocytic infiltrate, neutrophils, and eosinophils in the septae and surrounding adipose tissue [14]. Miescher's radial granuloma is characteristic of EN, consisting of small histiocytes surrounding a central cleft [15].

## 2 Case History/Examination

A 35‐year‐old Hispanic female with no significant past medical history presented to the emergency room with recurrent on‐and‐off episodes of fever, swelling, rash, and pain in her right thigh. Her symptoms had persisted for nearly 2 months and had led to multiple hospital visits where she was treated empirically for cellulitis (given fever, rash, tachycardia, and a computed tomography (CT) of her extremities showing soft tissue swelling suggestive of cellulitis). Each time, she was treated with intravenous antibiotics (initially treated with cefazolin and on subsequent visits treated with ceftriaxone and vancomycin), followed by oral antibiotics. Blood culture during each visit was unremarkable. In each visit, her symptoms got better in 3–4 days, but her symptoms recurred within a week of discharge. On her fourth visit, she again presented with fever, swelling, and a painful rash on her right thigh, accompanied by dizziness and nodular rashes on both thighs and shins. She denied taking any medications, having a family history of autoimmune diseases or cancer, or having a personal history of smoking, alcohol, or illicit drug use.

**FIGURE 1:**
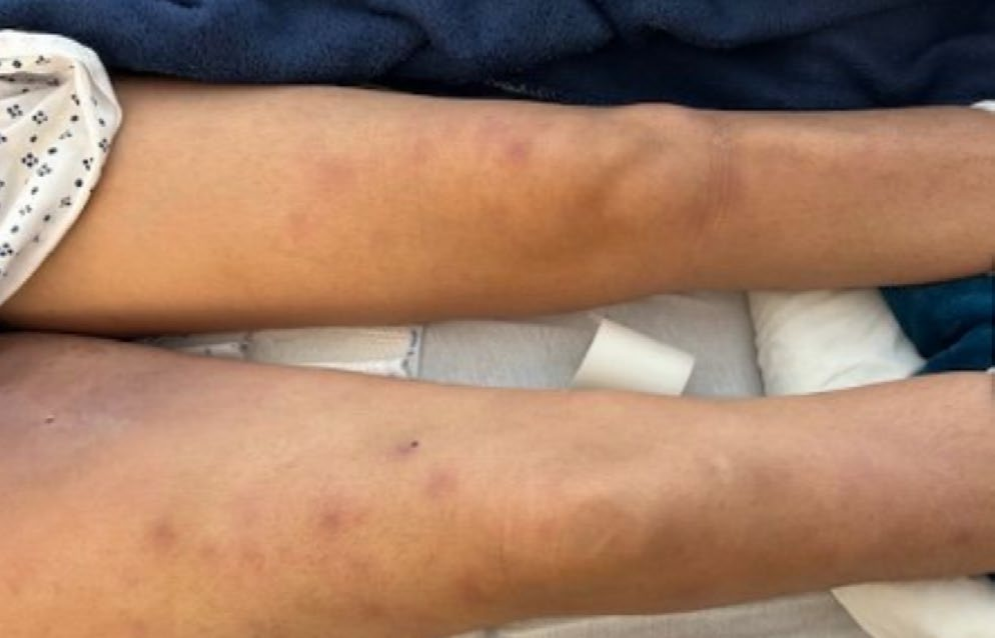
Erythema nodosum of the bilateral thigh.

On examination, she was tachycardic with a pulse rate of 120 beats per minute, a blood pressure of 95/57 mmHg, a temperature of 38.5°C, a respiratory rate of 20 breaths per minute, and a pulse oximetry reading of 98% on room air. She had also lost 5 pounds in the last 3 months. A clinical examination revealed significant induration and redness on the medial aspect of the right thigh, as well as multiple tender, erythematous nodular lesions on both legs (Figure 1). Both ankles were slightly swollen, with tenderness and limited range of motion.

## 3 Differential Diagnosis/Investigations

**FIGURE 2:**
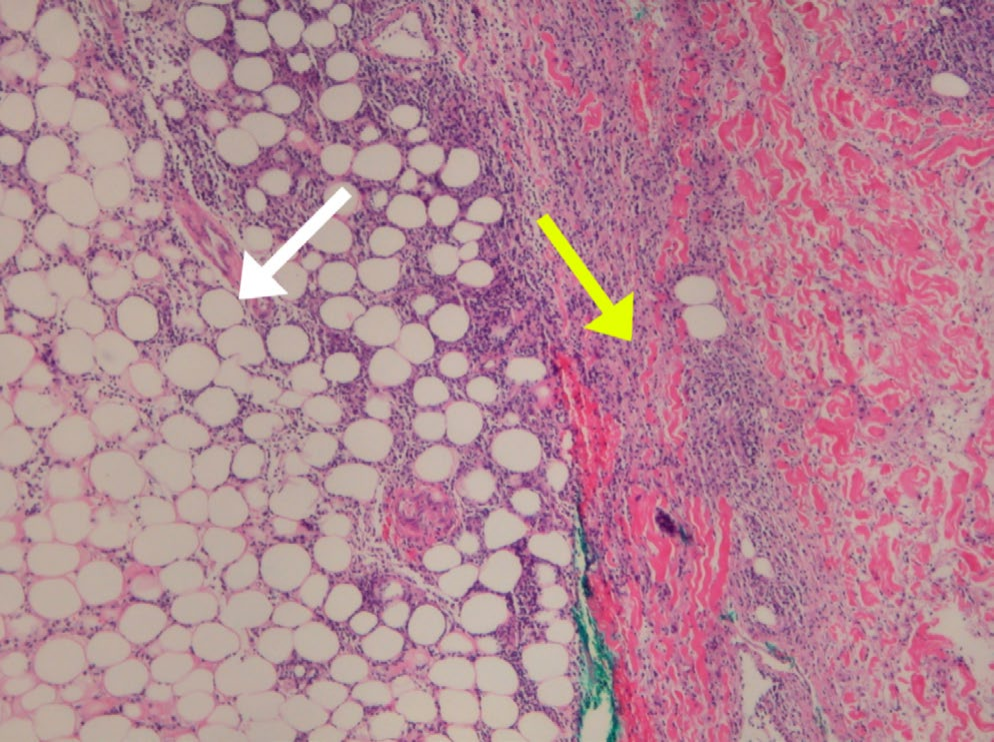
Histopathological images showing panniculitis (white arrow) and histiocytic infiltrates (yellow infiltrates).

**FIGURE 3:**
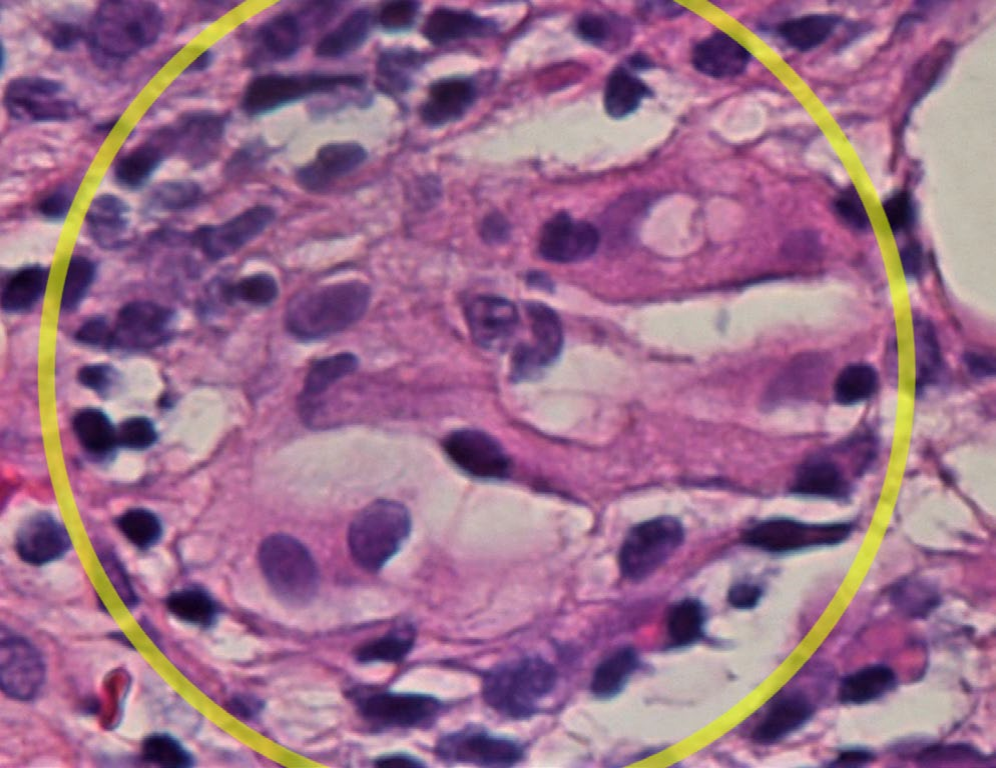
Magnified histopathological images of Meishner granuloma (yellow circle).

**FIGURE 4:**
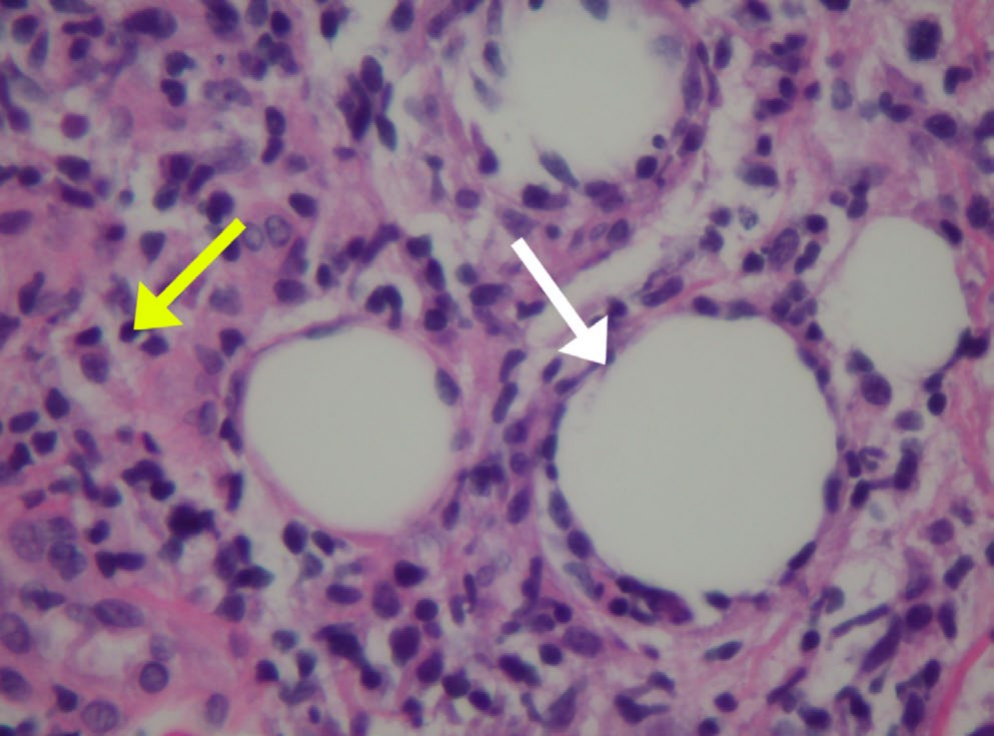
Magnified histopathological images of panniculitis (white arrow) and histiocytes (yellow arrow).

We considered broad differential diagnoses for this patient that include cellulitis, Lofgren syndrome, systemic lupus erythematosus (SLE), inflammatory bowel diseases (IBD), rheumatoid arthritis (RA), other autoimmune conditions, and infectious diseases, including fungal infections. Since she exhibited features of systemic inflammatory response syndrome (SIRS), initially thought to be secondary to cellulitis, she was started on broad‐spectrum antibiotics (piperacillin and tazobactam/vancomycin). She had experienced two spikes of fever in the first two days of admission. Despite not having leukocytosis and her recurrent admission for similar symptoms with no response to antibiotics, further workup was done. A skin biopsy was done, which showed septal panniculitis and histiocytic collection demonstrating intercellular clefting, suggestive of Miescher granuloma (Histopathological Figures 2, 3, 4). There was no evidence of vasculitis or atypia, and the spirochete stain was negative. AFB (acid fast bacilli) stain failed to highlight acid‐fast bacilli and fungal hyphae, respectively. Acknowledging that histologic changes are nonspecific, an extensive workup was done for erythema nodosum, considering infectious, autoimmune, and malignant etiologies.

Serologies for bacterial, mycobacterial, hepatitis profile, HIV, and fungal infections, including Aspergillus, Histoplasma, and Coccidioides, were sent, all of which returned negative. Antibody panel evaluations for autoimmune conditions revealed negative results for ANA, c‐ANCA, p‐ANCA, SSA, SSB, anti‐double‐stranded DNA, and anti‐Smith antibodies. Anti‐streptolysin O antibody screens, C3‐C4 levels, and total complement were also within normal ranges. Computed tomography of the chest did not show hilar or mediastinal lymphadenopathy. The transthoracic echocardiogram showed no evidence of vegetation. A colonoscopy with a random biopsy of the colonic mucosa revealed normal mucosa with no signs of inflammation, polyps, masses, or ulcers. However, the rheumatoid factor was elevated at 128, and the cyclic citrullinated peptide (CCP) antibody test revealed a strongly positive result of 80. These findings were suggestive of rheumatoid arthritis. X‐rays of the ankles and hands showed no fractures, dislocations, or soft tissue inflammation.

## 4 Conclusion and Results (Outcome and Follow‐Up)

After extensive evaluation, the patient was diagnosed with erythema nodosum secondary to rheumatoid arthritis. She was started on prednisone at a dose of 1 mg/kg, which resulted in rapid improvement of her symptoms, with resolution of fever, tachycardia, and rash within 48–72 h. The patient was discharged on a tapering dose of prednisone and was prescribed trimethoprim/sulfamethoxazole for *Pneumocystis jirovecii* pneumonia prophylaxis. Follow‐up with rheumatology showed continued improvement of the rash, and the patient was maintained on a low dose of prednisone (20 mg daily).

## 5 Discussion

Erythema nodosum (EN) can be caused by various etiologies, among which bacterial infections, inflammatory conditions, or malignancies are common. Streptococcal infection and sarcoidosis are known to cause this [8, 9, 10, 11, 12, 13, 14]. It is imperative to perform a thorough patient assessment, including history, physical examination, and laboratory evaluation, to reveal the etiology, as EN can be yet another manifesting symptom of an underlying disease process. EN can be identified as tender and erythematous skin lesions, especially nodules, located commonly on the pretibial surface of the lower extremities. It can also be present elsewhere in the extremities. These nodules can coalesce to form a plaque, as seen in our case. It can be diagnosed with a skin biopsy, which reveals septal panniculitis without vasculitis [14].

Our case presents a patient who initially had a fever and a rash on her right thigh for 2 months, which did not improve despite being treated with antibiotics on several occasions, until she was treated with steroids. The initial diagnosis of cellulitis was reasonable given the fever, rash, and tachycardia. Cellulitis usually presents with skin redness, warmth, subcutaneous induration, and systemic features including fever and chills. Not only cellulitis, but other forms of soft tissue infection have similar presentations. Since cellulitis can occasionally lead to complications like abscesses, necrotizing fasciitis, sepsis, or osteomyelitis, it is important to diagnose it early, which is mainly based on clinical features [16]. Although specific microorganisms cannot be isolated in most cases of cellulitis, it has been found that *Streptococcus pyogenes* and *Staphylococcus aureus* are the most common culprit bacteria [17]. As discussed earlier, EN can also occur secondary to streptococcal bacterial infection, but in our case, failure to improve despite several courses of antibiotics and recurrent fever does not support the diagnosis of cellulitis. Moreover, serologies for common bacterial and fungal infections were also negative. This shows the need to further investigate the cause of EN.

Löfgren syndrome is an acute form of sarcoidosis where one can have erythema nodosum, fever, acute arthritis, and bilateral hilar lymphadenopathy [18]. Chest imaging of our patient did not show hilar or mediastinal adenopathy. It is also important to rule out other autoimmune conditions associated with EN, including SLE. The patient denied recurrent oral ulcer and genital ulcer, and vascular ultrasound showed the absence of thrombosis in the deep venous system of the right lower extremity, making Bechet syndrome unlikely. Considering the patient had anorexia and weight loss with gastrointestinal discomfort, a colonoscopy with random colon biopsy was also done, with no signs of inflammation, polyps, masses, or ulcers, ruling out IBD. Previously, RA was simply diagnosed based on the presence of symmetric arthritis, but with the development of serological diagnostic tools, the American College of Rheumatology (ACR)/European League against Rheumatism (EULAR) shifted the diagnostic criteria from late‐stage features to early‐stage features along with the incorporation of serological abnormality [19]. This increased the sensitivity of the diagnosis of the disease [20, 21] and led to the diagnosis and treatment of the disease in its early stage, which can slow the progression. Anticitrullinated protein antibodies (ACPA) and rheumatoid factor (RF) are widely used serological markers for diagnosing RA. ACPA has higher specificity and sensitivity than RF for diagnosing RA, but may not correlate with disease activity [22, 23]. In our patient, an X‐ray image of the ankle or the hands did not show any hallmark changes of RA, including marginal erosions or soft tissue swelling. However, these findings may not be present in early RA, but they still can have seropositivity with ACPA [24, 25, 26]. RA can present with a wide variety of cutaneous manifestations, including rheumatoid nodules, neutrophilic dermatoses, and vasculitis, as in Felty syndrome and rheumatoid vasculitis, which are usually seen in patients with severe RA [6, 27]. Rheumatoid nodules are common skin lesions seen in patients with RA (14.7%–16.7% as per some studies) [28, 29]. However, RA can rarely manifest with erythema nodosum. There have been only a few reported cases of EN in patients with rheumatoid arthritis [30, 31]. A study of 130 patients by Karpova et al., who were referred with a diagnosis of erythema nodosum, showed that 35% were secondary to infectious causes and the other 35% due to Löfgren syndrome. Around 15% (20 patients) had EN secondary to rheumatic disease, out of which 2 (1.54%) were identified to have rheumatoid arthritis [32]. Treatment of erythema nodosum is largely based on etiology. Symptomatic treatment is aimed at alleviating pain and fever with anti‐inflammatory medications, including NSAIDs [8]. Steroids can be helpful once infectious causes or malignancies have been excluded and a preliminary diagnosis of an inflammatory disease, including rheumatoid arthritis, is made.

## Author Contributions

**Laxman Wagle:** conceptualization, formal analysis, investigation, resources, supervision, validation, visualization, writing – original draft, writing – review and editing. **Parmartha Basnyat:** conceptualization, formal analysis, methodology, resources, supervision, validation, writing – original draft. **Anuj Timshina:** conceptualization, formal analysis, supervision, validation, visualization, writing – original draft. **Rashmita Regmi:** conceptualization, formal analysis, resources, supervision, validation, visualization, writing – original draft, writing – review and editing.

## Ethics Statement

The authors have nothing to report.

## Consent

Written informed consent was obtained from the patient to publish this report in accordance with the journal's patient consent policy.

## Conflicts of Interest

The authors declare no conflicts of interest.

## Data Availability

All the required data are available in the manuscript itself.
